# P74 Addition of probenecid to β-lactam antibiotics in the treatment of skin and soft tissue infections: a systematic review

**DOI:** 10.1093/jacamr/dlag102.080

**Published:** 2026-06-26

**Authors:** Claudette Hewitt, Martin Llewelyn, Elizabeth Cross

**Affiliations:** Centre of Infection and Antimicrobial Research (CINAMR), Brighton and Sussex Medical School, University of Sussex, Falmer, UK; Department of Infection, University Hospitals Sussex NHS Foundation Trust, Brighton, UK; Centre of Infection and Antimicrobial Research (CINAMR), Brighton and Sussex Medical School, University of Sussex, Falmer, UK; Department of Infection, University Hospitals Sussex NHS Foundation Trust, Brighton, UK; Centre of Infection and Antimicrobial Research (CINAMR), Brighton and Sussex Medical School, University of Sussex, Falmer, UK; Department of Infection, University Hospitals Sussex NHS Foundation Trust, Brighton, UK

## Abstract

**Background:**

Among patients presenting to hospital with skin and soft tissue infection (SSTI), up to 50% have already failed oral antibiotic treatment and 90% receive IV antibiotics. As the NHS aims to shift care into the community, reducing the need for IV antibiotics is a priority. Probenecid, developed in the 1940s, prolongs serum concentrations of β-lactam antibiotics by delaying renal excretion. Its use is well established in gonorrhoea and syphilis but it could enable reduced use of IV and broad-spectrum agents in other indications such as SSTI. Probenecid-boosted oral treatment may be an alternative to IV for some patients. Alternatively, probenecid could allow less frequent dosing of narrow spectrum agents IV (e.g. cefazolin) reducing dependence on ceftriaxone in ambulatory settings.

**Objectives:**

This systematic review evaluated the effectiveness and safety of probenecid combined with β-lactam antibiotics for the treatment of SSTIs.

**Methods:**

MEDLINE and EMBASE were searched to February 2026. Eligible studies assessed the addition of probenecid to β-lactam therapy in human participants with SSTIs and reported clinical outcomes or adverse effects. Two reviewers independently screened studies, extracted data, and assessed risk of bias using RoB 2 for randomized controlled trials (RCTs) and ROBINS-I for non-randomized studies (NRS). Narrative synthesis was performed. PROSPERO: CRD420261290922.

**Results:**

Nine studies (three RCTs, six NRS) involving over 1600 participants were included, published between 1996 and 2024. Most studies evaluated once daily (OD) IV cefazolin plus probenecid versus ceftriaxone or more frequent cefazolin dosing. In adults, treatment success, failure, relapse, and hospital admission rates were similar between groups, across both RCTs and NRS. The only included paediatric study reported higher failure rates with more frequent cefazolin dosing compared with OD cefazolin plus probenecid, but this was based on a small subgroup analysis. One NRS compared OD IV cefazolin combined with probenecid with oral flucloxacillin plus probenecid and found no difference in failure rates, although this was a small retrospective study at serious risk of bias. One RCT compared OD IV cefazolin plus probenecid with oral cefazolin alone and found similar treatment failure rates, but excluded patients with prior antibiotic use, only followed patients to day 7, and was underpowered. Adverse events were not always systematically recorded and were inconsistently reported but were generally mild and predominantly gastrointestinal symptoms. Rates of adverse effects were similar in three studies, but where higher rates were reported, these were in the cefazolin plus probenecid group.

**Conclusions:**

Current evidence suggests that adding probenecid to β-lactam therapy may allow OD IV cefazolin to be used as an alternative to ceftriaxone for SSTI, supporting the use of narrower-spectrum antibiotics in ambulatory care settings. Evidence for using oral β-lactams plus probenecid in place of IV therapy remains limited. A well-designed RCT is needed to determine clinical non-inferiority and assess the acceptability of probenecid-related adverse effects. If shown to be effective, such an approach could reduce IV therapy complications, healthcare attendance, and overall costs.

